# Removal of an established invader can change gross primary production of native macroalgae and alter carbon flow in intertidal rock pools

**DOI:** 10.1371/journal.pone.0217121

**Published:** 2019-12-03

**Authors:** Francesca Rossi, Rosa M. Viejo, Linney Duarte, Fatima Vaz-Pinto, Ignacio Gestoso, Celia Olabarria

**Affiliations:** 1 Université Côte d'Azur, CNRS, UMR7035 ECOSEAS, Nice, France; 2 Área de Biodiversidad y Conservación, Universidad Rey Juan Carlos, Madrid, Spain; 3 MARE–Marine and Environmental Sciences Centre, Caniçal, Madeira Island, Portugal; 4 IIMAR/CIMAR,Centro Interdisciplinar de Investigação Marinha e Ambiental, Matosinhos, Portugal; 5 Departamento de Ecoloxía e Bioloxía Animal, Facultade de Ciencias del Mar, Universidade de Vigo, Vigo, Spain; University of Pavia, ITALY

## Abstract

The impact of invasive species on recipient communities can vary with environmental context and across levels of biological complexity. We investigated how an established invasive seaweed species affected the biomass, eco-physiology, carbon and nitrogen storage capacity of native seaweeds at sites with a different environmental setting due to a persistent upwelling in northern Spain. We removed the invasive Japanese wireweed *Sargassum muticum* from intertidal rock pools once every month during a one-year period and used an *in-situ* stable isotope pulse-chase labeling to estimate gross primary production (GPP), nitrogen uptake rate, ^13^C-carbon and ^15^N-nitrogen storage capacities. Following the addition of ^13^C-enriched bicarbonate and ^15^N-enriched nitrate to the seawater in the rock pools during the period of the low tide, we sampled macroalgal thalli at incoming tide to determine label uptake rate. After four days, we sampled macroalgal assemblages to determine both label storage capacity and biomass. After one year of removal there was no change in the macroalgal assemblage. However, both the GPP and ^13^C-carbon storage capacity were higher in the turf-forming *Corallina* spp. and, sometimes, in the canopy-forming *Bifurcaria bifurcata*. Nitrogen uptake rate followed similar, but more variable results. Although *S*. *muticum* inhibited carbon storage capacity of native species, the assemblage-level ^13^C-carbon storage was similar in the *S*. *muticum—*removed and control rock pools because the presence of the invasive species compensated for the functional loss of native species, particularly at sites where it was most abundant. No obvious effects were observed in relation to the environmental setting. Overall, the effect of the invasive *S*. *muticum* on carbon flow appeared to be mediated both by the effects on resource-use efficiency of native species and by its own biomass. Integrating physiological and assemblage-level responses can provide a broad understanding of how invasive species affect recipient communities and ecosystem functioning.

## Introduction

Biological invasion is an important component of global change, with a pervasive ecological impact on population, community and ecosystem functioning [1,2]. Invasive species impacts are context-dependent and variable in magnitude and direction, making their evaluation and prediction a difficult task [3,4].

When a non-indigenous species invades a community, native organism’s survival, physiological and ecological adaptations propagate to community and ecosystem processes through complex interactions among native and invasive organisms and with the environment [5–10]. Other components of global change such as warming and nutrient enrichment can also interact with the invasion, facilitate their success and change the impact on native species and ecosystem functioning [11–14]. Invasive species management should take into account the patterns of response over different biological scales and the interactions with other stressors in order to take adequate measures for controlling the impact at community and ecosystem levels [3]. Yet, very few studies on bioinvasions have considered the response at different levels of biological complexity simultaneously [3,5,15,16].

Carbon (C) and nitrogen (N) cycles are fundamental elements of ecosystem functioning and services. Eco-physiological and demographic traits such as photosynthetic rate, growth and biomass of both invasive and native primary producers are among the elements that can drive changes in ecosystem-based processes governing C and N cycles, such as productivity, C and N fixation, storage and mineralization [17,18]. A comparative approach linking changes in species traits to ecosystem processes, especially those related to C and N cycles could greatly contribute to improve management and control of invasions [3,5]. This can be worthy especially in the context of invasive ecosystem engineer species that can show strong ecological impacts of opposing directions on native populations, communities and ecosystem functions [4,13,14].

Marine coastal areas are particularly vulnerable to invasions due to numerous introduction vectors and activities that facilitate the spread of non-indigenous species [19]. In the last few decades, a large number of potentially highly invasive seaweeds have colonized marine coastal areas outside their range of distribution [20–23]. Invasion by the brown alga *Sargassum muticum* (Yendo) Fendsholdt is of particular concern, especially in Europe, where this species is classified as highly invasive [21]. The species has spread quickly across the European Atlantic coasts since its first appearance in the early 1970s. In the Iberian Peninsula, *S*. *muticum* became established as an invasive species in the 1980s [24,25]. *Sargassum muticum* is a canopy -forming macroalgal species that can efficiently compete for light and substrate availability and thus affect other macroalgae [26]. However, its ecological impact is very variable in magnitude and direction and there is still no consensus about the effects on recipient communities and ecosystem functions (see [27] for a review).

In this study we investigated if established populations of the invasive wireweed *S*. *muticum* affect biomass, eco-physiological traits associated with carbon (C) and nitrogen (N) flows and assemblage-level C and N storage of macroalgal assemblages. We also asked if the impacts are modified by the environmental setting. We removed the invader from selected intertidal rock pools once a month for more than one year in northern Spain (north of Galicia and eastern Asturias), within the broad transitional zones between cold- and warm-water [28]. Upwelling events occur in the northwest Spain and contribute increasing the differences in temperature and nutrient availability between eastern Asturias and northern Galicia [26,29,30].

In response to the complete removal of *S*. *muticum* from the rock pools, we expected to find an increase in the biomass of native species, in their capacity to fix and store C and N and also a change in the assemblage-level C and N pools. We also expected that the magnitude of the response would vary according to the cold-warm water transition zone and upwelling events.

We used an *in situ* stable isotope pulse-chase labeling experiment, involving the addition of ^13^C- and ^15^N- enriched inorganic compounds to the rock pools to measure any variations in the capacity of native species to fix and store nitrogen and carbon. Stable isotopes (SI) can be used at naturally occurring levels or can be used at levels outside the natural range by the addition of SI as tracers [31–33]. ^13^C-tracing can be used to estimate gross primary production, as an alternative to radioactive tracers (^14^C), the use of which is restricted in experimental studies [34,35]. Similarly, ^15^N tracing can be used to estimate the rate of nitrate or ammonium uptake [36,37]. The SI approach has been proposed as a tool to investigate plant physiology and the impacts that invasions can have on the food web of the recipient ecosystem [38,39]. However, to date studies on the use of SI as tracers in plant physiology have generally been conducted in the laboratory and application to field studies is at its infancy [31,40].

## Material and methods

### Study sites

**Fig 1:**
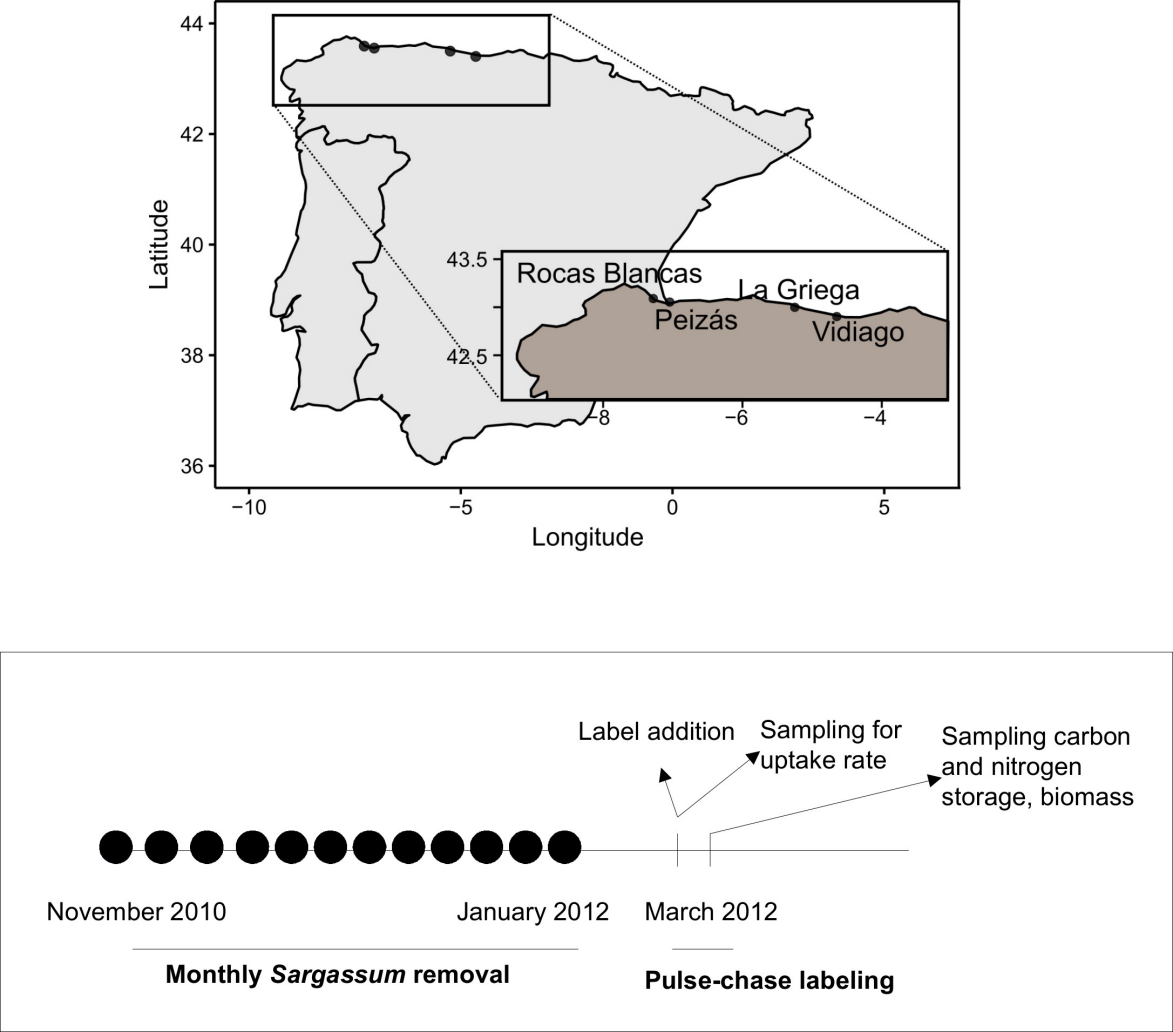
Study sites and sampling design. Map of the sites on the Spanish coast: Rocas Blancas (RB), Peizas (P), La Griega (LG), Vidiago (V) and timeline of the experiment.

Four sites were selected along the north Iberian Peninsula between the Atlantic and the inner part of the Biscay Bay (from 4° 39’ to 7° 15’ W). The sites had rocky platforms, which were semi-exposed to wave action, known to be invaded by *S*. *muticum*. Rocas Blancas (RB; 43° 33’ 21.91”N, 7° 2’ 31.42”W) and Peizas (P; 43° 35’ 23.95”N; 7°, 16’ 50.76”W) sites, situated in north of Galicia, receive cold, nutrient-rich upwelling water, whereas the sites at La Griega (LG; 43° 30’ 3.78”N, 5° 15’ 4.36”W) and Vidiago (V; 43° 24’ 8.17”N, 4° 38’ 58.14”W) in eastern Asturias are reached by warm waters from the Gulf current ([30]; Fig 1). We did not need permission or approval to access these sites because they are not under special protection.

The temperature of the oceanic water reaching the tidal rock pools was measured with field temperature data loggers (TidbitT® v2 Temp) placed in 2 rock pools at each site between November 2010 and September 2011. We recorded the temperature every 30 min and used two readings per day coinciding with incoming high tide to measure oceanic water temperature in the tide pools, without the effect of solar irradiation (minimal daily temperature in the pools). The concentration of inorganic nitrogen (NO_2_^-^ and NO_3_^-^) was measured by collecting 4 water samples at high tide on 6 dates between autumn 2010 and spring 2012 (November 2010, April-May 2011, June-July 2011, September 2011, April-May 2012, October 2012), except in Vidiago where sampling was not done in October 2011. The samples were frozen until analysis with a continuous flux autoanalyser.

### Experimental design and sampling

In November 2010, at each site we randomly selected 6 rock pools in the low intertidal area among those that were invaded by S. *muticum* and were of relatively similar size. The area and volume of the selected rock pools were determined by measuring the length, width and depth of each pool. Overall, the rock pools differed in area and volume among sites (ANOVA: F_3,16_ = 4.02, p = 0.03; F_3,16_ = 4.33, p = 0.02 for area and volume, respectively). The rock pools at Peizas and La Griega were on average the largest (P: area = 2.74 ± 1.24 m^2^ and volume = 0.75 ± 0.16 m^3^; LG: area: 2.31 ± 1.16 m^2^ and volume: 0.41 ± 0.11 m^3^). The Rocas Blancas and Vidiago rock pools were the smallest (RB: area = 1.33 ± 0.62 m^2^ and volume = 0.22 ± 0.04 m^3^; V: area = 1.05 ± 0.29 m^2^ and volume = 0.26 ± 0.07 m^3^). However, *a posteriori* test SNK (p = 0.05) did not detect any significant difference between sites.

Between November 2010 and February 2012, we removed *S*. *muticum* every month by hand from half of these rock pools (hereafter *S*. *muticum—*removed, -S) at each site. The remaining rock pools were left undisturbed (hereafter control pools, +S). There were no differences in size among the *S muticum—*removed and the control rock pools (ANOVA, F_1,16_ = 0.74 p = 0.40 and 0.48, p = 0.50 for area and volume, respectively).

At the beginning of the spring tide (10^th^ March 2012), we added K^15^NO_3_ (99% of ^15^N atoms, SIGMA) and NaH^13^CO_3_ (99% ^13^C atoms, SIGMA) to the water remaining in the tide pools (Fig 1 for the timeline of the experiment). We added 0.006 mg of ^15^N L^-1^ and 0.52 mg of ^13^C L^-1^ to each rock pool, varying the amount of label according to the volume of the pool. These concentrations were expected to increase the heavy isotope atoms in the water by up to 2% of the total C or N, assuming a concentration of dissolved inorganic carbon (DIC) of 2 mmol L^-1^ and that of total nitrogen (NO_2_^-^, NO_3_^-^ and NH_4_^+^) of 0.02 mmol L^-1^. The amounts were calculated before the analytical data on nitrogen concentration in seawater were available. These data were incorporated in the estimates of C and N fixation and storage, as shown in the next section. We used low isotope-addition levels to prevent altering the natural biogeochemistry of the seawater (nitrogen enrichment or acidification of the seawater). During the incoming tide (day 1; 2–3 h after addition of label), we sampled small amounts of the dominant seaweeds to assess the amount of label they could fix during exposure. The tidal rock pools position (low intertidal and semi-exposed sites) allowed seawater renewal every tidal cycle. Therefore, the label not fixed during low tide would be washed away by incoming tide, as previously observed in other systems [41,42]. The fixed label would be instead partly stored in the thalli and partly released by seaweed metabolism (respiration, ammonium release). Four days after adding the label, we sampled seaweed biomass and isotope incorporation. At the sites in Galicia (Peizas and Rocas Blancas), we also took samples at intermediate times (24 and 48 hours; day 2 and 3, respectively). On day 2, only the Peizas site was sampled as a local storm prevented sampling at Rocas Blancas. During the final sampling, 20 × 20 cm quadrats were randomly selected in each rock pool. The number of quadrats per pool varied from 2 to 5, according to the pool size. The total number of quadrats necessary to cover the pool surface was visually estimated in the field. The seaweeds within the quadrats were removed and transported to the laboratory, identified to the lowest taxonomic level possible, dried at 60°C for 48h and weighed. We also sampled seaweeds outside the tested rock pools to determine background isotopic values.

We expressed macroalgal biomass per sampling unit (0.04 m^2^) and per experimental unit (rock pool). The former indicated the localized macroalgal biomass, independently of the size of the pools. Unless otherwise, results did not differ and we only report those for macroalgal biomass per rock pool, as for the carbon and nitrogen flows (see below).

After collecting the samples, we selected which species to use for stable isotope analysis. *Bifurcaria bifurcata* R. Ross and *Corallina* spp. were the dominant taxa at all sites, making up 70% of the biomass of native species. These species were also present in the majority of tide pools across the four sites, thus enabling the consistency of the effect of *S*. *muticum* to be tested across sites. In addition, they represented two different morphological and functional groups, as *B*. *bifurcata* is a canopy-forming brown seaweed, and *Corallina* spp. is a primary space holding, turf-forming red seaweed. We also selected *Ceramium* spp., a small sub-canopy forming seaweed. Although it was not particularly abundant, it was widespread across the four sites, thereby allowing cross-site comparisons.

### Isotopic analysis and calculations

The C and N concentrations and stable isotopic composition (δ^13^C and δ^15^N) were measured in the dried and grounded samples of the selected dominant native species with a Fisons elemental analyser coupled on line via a Finningan conflo 2 interfaces, to a Finningan delta S mass spectrometer. The isotope ratios are expressed in the delta notation (δX = δ^13^C or δ^15^N) and in units of ‰, as in Eq 1. Reference values are based on the isotopic composition of Vienna PDB for C and atmospheric nitrogen for N.

$$\delta \text{X}=\left[\left(\text{Rsample}/\text{Rreference}\right)-1\right]\times 10^{3}$$ (1)

The excess of heavy isotopes (E) was estimated above background values as % atoms (the proportion of heavy isotope (in weight of atoms) over the total carbon or nitrogen), as derived from Eq 1.

$$\text{E}=\%{\text{atomsX}}_{\text{end}}-\%{\text{atomsX}}_{\text{background}}$$ (2)

Gross primary production (GPP) and nitrogen uptake rate were calculated from the amount of label assimilated during the first tidal cycle (2–3 hours of exposure). GPP was derived using the equations by [Miller and Dunton 2007] and normalised to the dry weight of the macroalgae in the pool (DW in Eq 3). Nitrogen uptake rate followed the equation proposed by [Naldi and Wheeler 2002], under the assumption that there was no change in the macroalgal biomass during the hours of exposure to the label (Eq 4):

$$\text{GPP}\left({\text{mgCg}}^{-1}{\text{drywth}}^{-1}\right)=\left(\text{OCthalli}\times \text{Ethalli}\right)/\left(\text{tincubation}\times \text{Ewater}\right)/\text{DW}$$ (3)

$$\text{Nitrogen uptake rate}\left({\text{mgNg}}^{-1}{\text{drywth}}^{-1}\right)=\left(\text{Nthalli}\times \text{Ethalli}\right)/\left(\text{tincubation}\times \text{Ewater}\right)/\text{DW}$$ (4)

where C and N indicate the mg of ^13^C-Carbon or ^15^N-nitrogen and t is the time of exposure.

We then estimated the role of seaweeds in sequestrating C and N at the end of the study period as net mg ^13^C-Carbon or ^15^N-nitrogen incorporations.

mgX=E×OC,N (5)

As the amount of isotopes added was proportional to the volume of the pools and therefore differed between pools, the data were expressed as the percentage of total mg ^13^C-Carbon or ^15^N-nitrogen added to the pool.

### Statistical analysis

We analyzed the response of the native assemblage to the removal of *S*. *muticum* using PERMANOVA (999 permutations) on the triangular matrix created by pairwise Bray-Curtis index after log transformation of biomass data to decrease the relative importance of most abundant species [43]. The biomass of *S*. *muticum* was excluded from this analysis because it was manipulated as part of the design. According to the experimental design, the statistical model included two orthogonal fixed factors: Site (4 levels) and *S*. *muticum* presence (2 levels) with 3 replicate pools. Site was considered a fixed factor because sites were selected on the basis of the presence of local upwelling events and established presence of *S*. *muticum*. *A posteriori* pairwise comparisons were done when significant differences were detected in the *S*. *muticum* × Site interaction term or in the main term Site.

Analysis of variance (ANOVA) and generalised least square (GLS) models were used to test for differences in the total number of species and in the biomass of native macroalgae (whole assemblage, canopy and dominant species), as well as in the gross primary production, nitrogen uptake rate, ^13^C-carbon and or ^15^N-nitrogen incorporation. The GLS model considered residual variances correlated to the mean values of sites or *S*. *muticum* × Site interaction term (model varIdent in [44]). The optimal model was then selected using Akaike's Information Criteria, AIC [44]. When the optimal model indicated significant differences for *S*. *muticum* term, we used *a posteriori* comparisons to detect at which sites the removal of *S*. *muticum* had an impact. When the ANOVA yielded the best model, we used the SNK (Student-Newman-Kuels) test for *a posteriori* multiple comparisons. Otherwise, we used pairwise least-square means comparisons using t-test. We analysed the temporal changes of excess (E) above background with repeated measure linear-mixed model (GLM) [45]. Site and *S muticum-*removed factors were included in the analyses for native species as fixed and orthogonal, while rock pool was included as random factor, nested in the interaction and sampled at different repeated times. For the excess label measured in *S*. *muticum*, we used a GLM model with one fixed factor (Site) and rock pool nested in Site as random factor.

We used ANOVA with Month and Site as fixed and orthogonal factors to test for differences in temperature among sites. We run separate analyses for each season. For seawater nutrient analyses we used ANOVA model with Time of sampling and Site as fixed factors.

Assumption of linearity and homogeneity of residual variances (when necessary) were checked by inspecting plots of residuals vs fitted values. If these conditions were not found and the VarInd structure did not correct residual variance heterogeneity, we log-transformed data, as indicated in the result section. All analyses were done in R 3.2.3 using the libraries nlme for the GLM and GLS model, lme4 and lsmeans for pairwise comparison tests and GAD for analyses of variance and *a posteriori* SNK test (R Development Core Team 2011). Multivariate analysis was run using PRIMER v.6.0 +PERMANOVA.

## Results

### Environmental setting

Temperatures were similar during autumn and winter and started to differentiate in spring. The largest differences among sites were in summer (S1 Fig). In northern Galicia, the sites had oceanic water in the rock pools of about 1°C lower than in eastern Asturias (V: 19.66 ± 0.08°C, LG: 19.54 ± 0.08°C, P: 18.73 ± 0.07°C, RB: 18.25± 0.08). The analyses showed significant differences in the Month × Site interaction term, both in spring and summer (ANOVA, F_6,684_ = 11.26 p < 0.001; F_6,684_ = 4.23, p < 0.001, respectively). The *a posteriori* SNK test showed that in spring differences among sites occurred in May only, when La Griega (LG) and Rocas Blancas (RB) sites had the warmest and coldest seawater temperatures, respectively. In summer, the eastern Asturias sites (LG and Vidiago, V) had the highest temperature and at Peizas (P) seawater temperature was warmer than at Rocas Blancas (V = LG > P > RB). The differences among sites in seawater nutrient concentration varied through time and sites (S1 Fig; ANOVA for the Time × Site interaction term: F_15,72_ = 4.76, p< 0.001; data were log-transformed). The SNK test showed *a posteriori* that in November 2010 Rocas Blancas had the highest values and that Peizas had the lowest values in April-May 2012, just after the labeling experiment was carried out (March 2012).

### Isotopic enrichment experiment

The excess E (Eq 2) of ^13^C-carbon and ^15^N-nitrogen in native seaweed thalli did not decrease during the 4 days of the experiment (S2 Fig) and values were lower in the control than in the *S*. *muticum—*removed pools at the Peizas site (P in Table 1). The AIC comparisons showed no differences between auto-correlative and repeated-measure GLM models for either carbon or nitrogen. Excess label in *S*. *muticum* did not differ between sites (S2 Fig).

**Table 1:** Parameter estimates and standard errors of the GLM models for the excess E of ^15^N and ^13^C atoms in native macroalgae.

(a) Fixed effect “*S*. *muticum*”							
		***B*. *bifurcata***		***Corallina* spp.**		***Ceramium* spp.**	
		**% ^15^N**	**% ^13^C**	**% ^15^N**	**% ^13^C**	**% ^15^N**	**% ^13^C**
**P**	**-S**	17.52 (2.97)*	31.59 (3.01)*	20.44 (2.87)*	54.26 (7.25)*	29.42 (9.02)	70.00 (11.17)*
	**+S**	8.27 (2.97)	10.67 (3.01)	9.86 (2.46)	13.76 (7.24)	15.91 (9.22)	26.47 (11.48)
**RB**	**-S**	10.03 (2.94)	10.58 (3.32)	13.84 (2.61)	23.06 (7.66)	39.56 (9.25)	46.11 (11.28)
	**+S**	7.61 (2.94)	10.34 (3.32)	9.72 (2.87)	15.13 (7.79)	21.68 (8.29)	39.67 (10.06)
**LG**	**-S**	9.12 (3.52)	9.92 (3.44)	10.00 (3.23)	13.41 (8.32)	33.60 (9.46)	27.17 (11.19)
	**+S**	11.87 (3.99)	12.11 (3.44)	9.24 (5.16)	19.83 (10.93)	30.68 (10.91)	38.20 (12.24)
**V**	**-S**	7.74 (3.52)	10.32 (3.44)	10.95 (3.23)	23.15 (8.32)	15.77 (17.59)	30.51 (16.99)
	**+S**	7.25 (3.52)	13.03 (3.44)	7.10 (3.66)	19.97 (8.99)	23.05 (18.03)	32.81 (18.68)
(b) Random effects							
		**%** ^**15**^**N**	**%** ^**13**^**C**	**%** ^**15**^**N**	**%** ^**13**^**C**	**%** ^**15**^**N**	**%** ^**13**^**C**
**Rock pool**		0.16	12.17	11.49	16.63	32.11	34.61
**Days**		2.31	3.28	5.58	1.39	15.73	14.59
**Residual**		7.00	5.51	7.70	14.70	6.48	13.69

*: Significant differences between the 2 levels of the fixed factor.

Table 1 legend. (a) Mean (SE) estimates from the GLM model for the removal of *S*. *muticum* at each site (P, RB, LG, V, as indicated in Fig 1 legend) for the fixed-effect *S*. *muticum* (2 levels: no *S*. *muticum*: -S; control: +S); (b) Standard deviation of the random effect “days” (repeated measure within replicate pools).

### Macroalgal assemblage

At the time of sampling, native macroalgal assemblages did not differ following the removal of *S*. *muticum* and this pattern was consistent across sites (PERMANOVA: F_1, 16_ = 1.30, p = 0.24 and F_3,16_ = 0.88, p = 0.30, for the term *S*. *muticum* and the *S*. *muticum* × Site interaction term, respectively; S3 Fig). The total number of species and their biomass showed a similar pattern of variation (Table 2). The biomass of each of the dominant species per rock pool did not differ between *S*. *muticum—*removed and control rock pools, and this pattern was consistent across sites (Table 2). The biomass of native canopy-forming species was dominated by *B*. *bifurcata* (90% of native canopy biomass across sites; Table 2) and, as for this species canopy-forming biomass did not differ in relation to the removal of *S*. *muticum* (Table 2).

**Table 2:** Diversity and biomass of the macroalgal assemblages.

						Summary ANOVA	
		P	RB	LG	V	*S*. *m*.	*S*. *m*. × Site
**No. of** **species**	+S	9.3(0.7)	9.7(0.9)	5.0 (0.0)	3.7 (0.3)	F_1,16_ = 3.06 p = 0.10	F_3,16_ = 2.38 p = 0.11
	-S	8.0(0.6)	6.3 (1.3)	5.0 (1.0)	4.3(0.9)		
**Native** **Assemblage**	+S	1302.4 (439.1)	912.6 (134.2)	749.1 (297.0)	549.2 (107.8)	F_1,16_ = 0.14 p = 0.72	F_3,16_ = 0.19 p = 0.90
	-S	991.8 (73.6)	853.6 (118.1)	753.1 (488.7)	629.2 (171.6)		
***B*.** ***bifurcata***	+S	519.7 (297.5)	433.0 (56.3)	301.8 (119.9)	392.8 (83.0)	F_1,16_ = 1.77 p = 0.20	F_3,16_ = 0.65 p = 0.60
	-S	247.8 (32.6)	360.9 (114.6)	143.9 (50.1)	499.4 (152.1)		
***Corallina*** **spp.**	+S	431.8 (82.7)	283.0 (90.2)	67.9 (34.0)	8.4 (4.2)	F_1,16_ = 2.54 p = 0.13	F_3,16_ = 0.32 p = 0.81
	-S	519.1 (135.5)	229.2 (78.7)	147.7 (69.1)	32.3 (7.4)		
***Ceramium*** **spp.**	+S	20.7 (15.8)	10.4(6.6)	5.6 (0.7)	0.7 (0.7)	F_1,16_ = 0.55 p = 0.47	F_3,16_ = 1.92 p = 0.17
	-S	1.8 (1.6)	5.0(4.0)	7.0 (4.3)	7.3 (4.8)		
**Native** **Canopy**	+S	568.2 (342.6)	434.4 (57.4)	315.3 (112.8)	392.8 (83.0)	F_1,16_ = 0.36 p = 0.55	F_3,16_ = 0.20 p = 0.89
	-S	324.4 (91.5)	361.9 (115.6)	255.9 (93.4)	501.5 (152.1)		
***S*.** ***muticum***	+S	868.7 (375.1)	16.3 (14.9)	95.9 (34.8)	52.7 (21.0)		
	-S	15.6 (9.6)	0.7(0.7)	0.0 (0.0)	0.0 (0.0)		

Table 2 legend. Mean (SE) number of native species (No. of species) and the biomass (g dw pool^-1^) of the native macroalgal assemblage, of the dominant and most represented macroalgae used for isotopic analyses, of native canopy-forming macroalgae and of the invasive species (*S*. *muticum*) at each of the four sites (P, RB, LG, V, as indicated in Fig 1 legend). Values are averaged over the 3 rock pools where *S*. *muticum* was removed (-S) or the control rock pools (+S). Summary of analyses of variance (ANOVA) with F ratio and significant p values for the *S*. *muticum* term (*S*. *m*.) and the interaction term *S*. *muticum* × Site is also indicated. Biomass data were log-transformed.

We observed different macroalgal assemblages between sites, independently of the removal of *S*. *muticum* (PERMANOVA; F_3, 16_ = 4.97, p < 0.001). There were more species at Peizas and Rocas Blancas than at La Griega and Vidiago (ANOVA: F_3,16_ = 15.74, p < 0.0001; SNK test: P = RB > LG = V, p < 0.05, Table 1). Overall, species biomass did not vary (F_3,16_ = 1.49, p = 0.25; F_3,16_ = 0.77, p = 0.53; F_3,16_ = 2.43, p = 0.10, F_3,16_ = 0.79, p = 0.52 for all species, for *Ceramium* spp. and *B*. *bifurcata* and for canopy-forming macroalgae, respectively; Table 2), except that of *Corallina* spp. (F_3,16_ = 13.88 p = 0.0001, SNK test: P > RB = LG > V, p < 0.05). However, when we considered biomass per sampling unit (0.04 m^2^), the biomass of canopy-forming species was greater at Peizas and La Griega than at the remaining sites (SNK test: V: 16.2 ± 2.1 = RB: 15.1 ± 3.3 > P: 8.0 ± 1.7 = LG: 6.7 ± 0.7; p < 0.05).

In the control rock pools, *S*. *muticum* biomass differed among sites, but the *a posteriori* multiple comparison SNK test did not identify any alternative pattern (ANOVA for sites: F_3,8_ = 8.05, p = 0.01; Table 1). Analysis of *S*. *muticum* biomass per sampling unit (0.04 m^2^) revealed that biomass was greater at Peizas than at any other site (ANOVA: F_3,8_ = 12.36, p = 0.002; SNK-test: P > LG = RB = V, p < 0.05). When the biomass of canopy, habitat-forming native species was combined with that of *S*. *muticum*, biomass was found to be significantly greater in control than in the *S*. *muticum—*removed pools (F_1, 16_ = 4.90, p = 0.01), consistently across sites.

### Eco-physiological response

**Fig 2:**
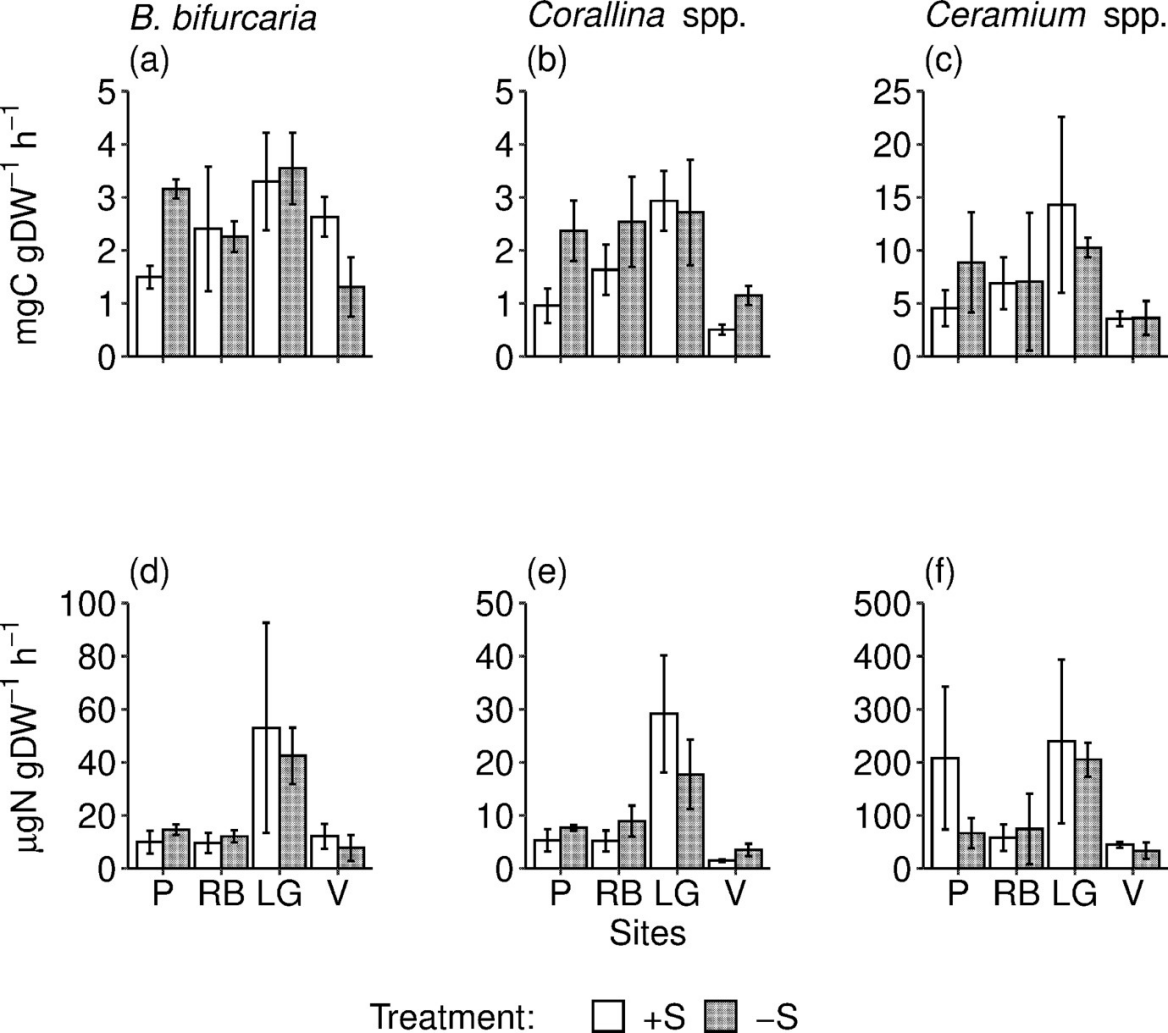
Estimates of macroalgal physiological traits. Mean (SE) gross primary production (a-c) and nitrogen uptake rate (d-f) of the 3 dominant native macroalgae in *S*. *muticum—*removed (-S) and control rock pools (+S) at each site (P, RB, LG, V, as indicated in Fig 1 legend).

The gross primary production (GPP) was lower in individuals of *Corallina* spp. and *B*. *bifurcata* collected in the control (+S) than in the rock pools where *S*. *muticum* was removed (-S). This pattern was consistent across sites for the former species, but it was significant only at the Peizas site for the latter species. *Ceramium* spp. did not show any significant effect (Fig 2A–2C and Table 3). The pattern of response to the removal of *S*. *muticum* for the rate of nitrogen uptake was similar to that of GPP, but we did not identify any significant change (Table 3 and Fig 2D–2F). The GPP and nitrogen uptake rate of *S*. *muticum* did not vary significantly between sites (GLS2 model; F_3,8_ = 0.48, p = 0.70 for GPP; F_3,8_ = 1.31, p = 0.34 for nitrogen uptake rate after log-transformation).

**Table 3:** Parameter estimates of the GLS models and t-tests for gross primary production and nitrogen uptake rate of the native macroalgae.

	*B*. *bifurcata*		*Corallina* spp.		*Ceramium* spp.	
	-S *vs* +S Mean (SE)	t	-S *vs* +S Mean (SE)	t	-S *vs* +S Mean (SE)	t
**Gross Primary Production**						
	Fit: GLS2		Fit: GLS2_red		Fit: ANOVA_red^^^	
**P**	1.66 (0.28)	5.94*	0.69(0.18)	3.73*	0.09 (0.22)	0.79
**RB**	-0.14(1.21)	-0.12				
**LG**	0.25 (1.14)	0.22				
**V**	-1.32(0.67)	-1.97				
**Nitrogen uptake rate**						
	Fit: ANOVA ^^^		Fit:ANOVA ^^^		Fit: ANOVA_red ^^^	
**P**	0.34 (0.68)	0.51	0.61 (0.53)	0.27	0.44 (0.45)	0.97
**RB**	0.33 (0.68)	-0.49	-0.44 (0.53)	0.42		
**LG**	0.52 (0.68)	0.77	0.52 (0.53)	0.97		
**V**	-0.61 (0.68)	-0.89	0.78 (0.53)	0.17		

*: p<0.05

^: log-transformed data

Table 3 legend. The t-test on least-square mean estimates between *S*. *muticum—*removed (-S) and control (+S) rock pools at each site (P, RB, LG, V, as indicated in Fig 1 legend). “Fit” indicates the best-fit model (ANOVA: no variance structure; GLS2: residual variance varies with mean values of sites). When the interaction term of the best-fit model was not significant (p > 0.25), we used a reduced model with only the main factors (“_red” in Table).

**Fig 3:**
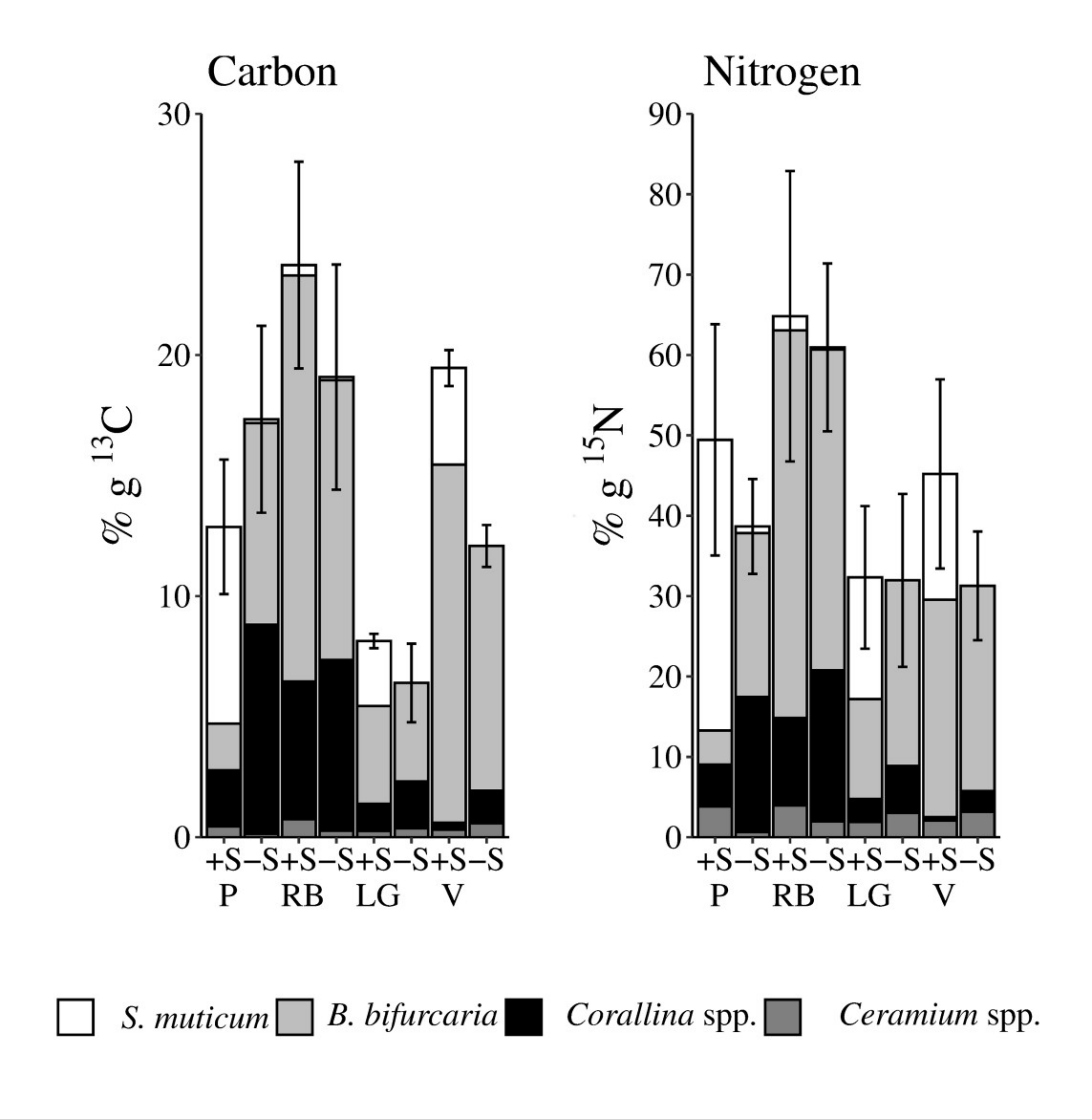
Macroalgal carbon and nitrogen pools. Mean (SE) mg ^13^C-carbon and ^15^N-nitrogen retained by the assemblage of the 3 native species (*B*. *bifurcata*, *Corallina* spp and *Ceramium* spp.) and *S*. *muticum*. The average contribution of each species to these pools is indicated in different colors. P, RB, LG, V, as indicated in Fig 1 legend.

The mg of ^13^C-carbon and ^15^N-nitrogen stored in the thalli also varied according to the removal of *S*. *muticum*. The native species *B*. *bifurcata* and *Corallina* spp. retained significantly more carbon and nitrogen in -S than +S rock pools at Peizas and consistently across sites, respectively. *Ceramium* spp. stored more nitrogen in +S pools at Peizas (Table 4 and Fig 3). The overall ^13^C-carbon stored in the three native species was significantly higher in -S than in +S rock pools at Peizas site. These differences disappeared when the ^13^C-carbon stored in *S*. *muticum* was included in the estimate (Table 4 and Fig 3). There was also an increase of the ^13^C-carbon stored in the +S as compared to the -S rock pools at the site of Vidiago (Fig 3). The ^15^N-nitrogen pool in both the assemblages of the three native species and of the three native + invasive species showed a the same trend observed for the ^13^C-carbon (Fig 3). However, the statistical analysis did not identify any significant effect, probably due to high variability between rock pools (Table 4). The concentration of both ^13^C-carbon and ^15^N-nitrogen in *S*. *muticum* varied among sites (ANOVA model; F_3,8_ = 4.79, p = 0.03; 4.84 p = 0.03 for ^13^C and ^15^N, respectively; data were log-transformed). *A posteriori* SNK test (p < 0.05) did not identify any clear difference, although the accumulation was particularly low at Rocas Blancas (Fig 3).

**Table 4:** Parameter estimates of the GLS models and t-tests for mg of ^13^C-carbon and of ^15^N-nitrogen stored in thalli.

	*B*. *bifurcata*		*Corallina* spp.		*Ceramium* spp.		Native species		Native+*S*. *muticum*	
	-S *vs* +S Mean (SE)	t	-S *vs* +S Mean (SE)	t	-S *vs* +S Mean (SE)	t	-S *vs* +S Mean (SE)	t	-S *vs* +S Mean (SE)	t
**Carbon pool**										
*Fit*	GLS3		ANOVA_red^^^		ANOVA_red		ANOVA		GLS2	
**P**	6.42 (2.55)	2.52*	0.46 (0.18)	2.49	-0.44 (0.45)	-0.97	10.68 (4.15)	2.57*	2.68 (1.66)	0.58
**RB**	-5.23(6.40)	-0.82					-4.34(4.15)	-1.05	-4.65(6.34)	0.47
**LG**	0.02 (1.66)	0.01					0.06(4.15)	0.01	-2.64(1.66)	0.13
**V**	-4.71(3.55)	-1.33					-4.57 (4.15)	-1.11	-8.57(1.15)	-7.48
**Nitrogen pool**										
*Fit*	ANOVA^^^		ANOVA_red^^^		ANOVA^^^		ANOVA		ANOVA	
**P**	1.31 (0.42)	3.12*	0.72 (0.18)	3.86	-0.95 (0.42)	-2.31	21.30 (13.39)	1.59	-13.99(16.24)	-0.86
**RB**	-0.11 (0.42)	-0.27			-0.39 (0.42)	-0.93	-2.39 (13.39)	-0.18	-3.88 (16.24)	-0.24
**LG**	1.31 (0.42)	1.35			0.34 (0.42)	0.81	12.90 (13.39)	0.35	-2.26 (16.24)	-0.14
**V**	0.02 (0.42)	0.05			0.19 (0.42)	0.47	-0.97 (13.39)	-0.07	-16.62 (16.24)	-1.02

*: p<0.05

^: log-transformed data

Table 4 legend. The t-test was applied to the least-square mean estimates for *S*. *muticum* term (-S vs. +S) at each site (P, RB, LG, V, as indicated in Fig 1 legend). Results refer to the best-fit model (*Fit*), which varied among the response variables and was selected using AIC among the ANOVA and GLSs models (GLS2 = residual variance related to sites; GLS3 = residual variance related to *S*. *muticum* × Site interaction term). When the interaction term of the best-fit model was not significant (p > 0.25), we used a reduced model with only main factors (“_red” suffix).

**Fig 4:**
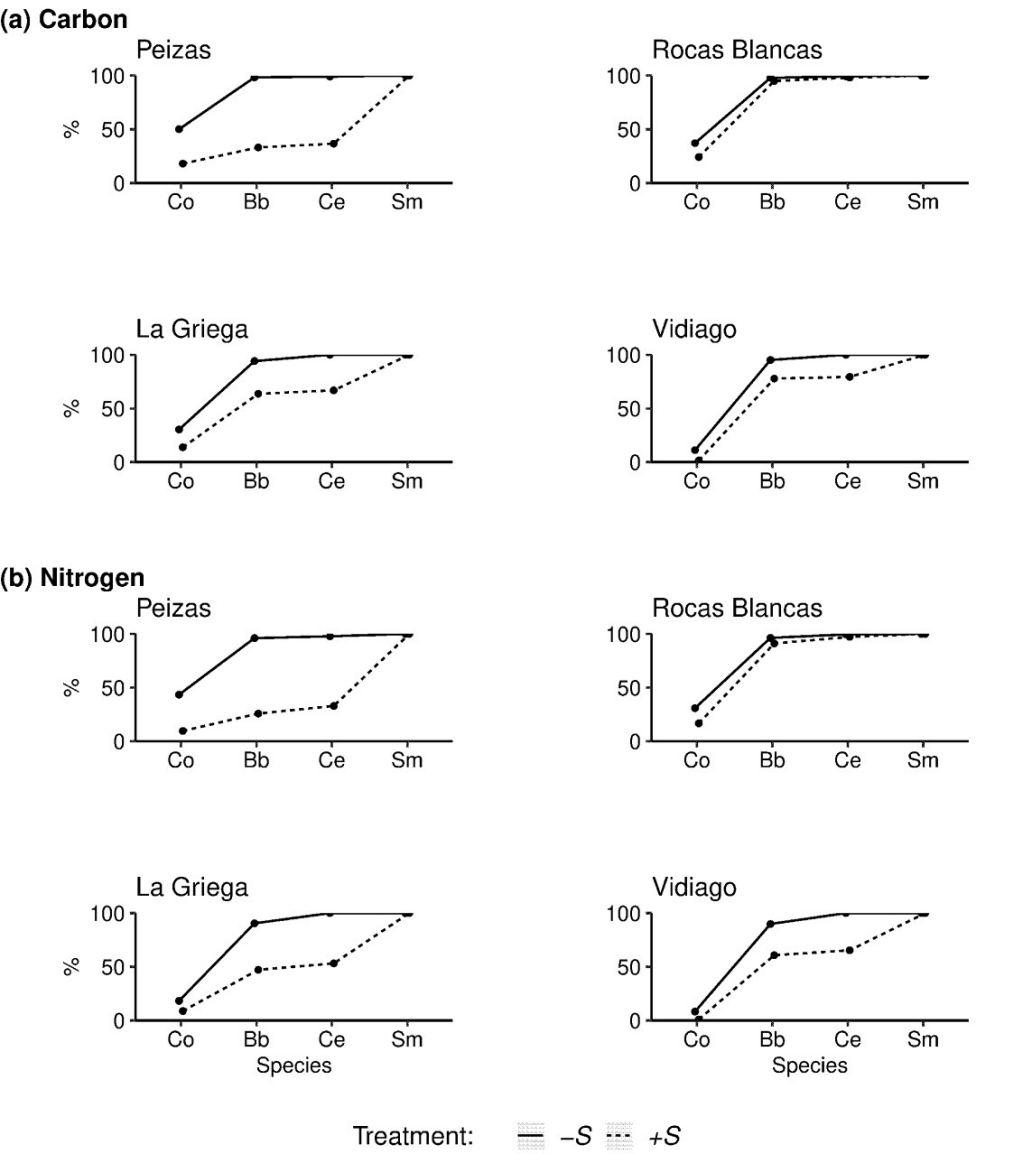
Cumulative contribution to carbon and nitrogen pools. Average percentage mg of ^13^C-carbon (a) and of ^15^N-nitrogen (b) of the native species (Co = *Corallina* spp., Bb = *Bifurcaria bifurcata*, Ce = *Ceramium* spp.) and of the invader *Sargassum muticum* (Sm) in control (+S) and *S*. *muticum—*removed (-S) rock pools at each site.

The cumulative % contribution to the ^13^C-carbon and ^15^N-nitrogen pools of native species (*Corallina* spp. and *B*. *bifurcata)* was higher in *S*. *muticum—*removed (-S) than control (+S) rock pools. The cumulative % of these two native species and *S muticum* in the +S was comparable to that of the two native species in the -S rock pools, except at Rocas Blancas where *S*. *muticum* contribution was not relevant (Fig 4).

## Discussion

Our experiment was designed to detect if the impact of the removal of the invasive seaweed *Sargassum muticum* from rock intertidal pools could vary with the environmental setting relate to an upwelling current that can brings nutrient-rich, cool seawater nearby the coast of north of Galicia [29]. Although there were no differences in daily average temperature when this study was done (March 2012), daily average temperature diverged in May and in summer. And the range of temperature was always larger at the sites in Asturias than at those in Galicia all-year-around during 2011 [30]. Moreover, air temperature and number of sun hours that differ among these regions can further affect the seawater temperature in rock pools during low tide [30]. Seawater nutrient concentration, instead, showed large spatial and temporal variability in agreement with the presence of local anthropogenic nitrogen sources rather than upwelling-driven nutrient concentrations all-year-around [46]. Therefore, the initial hypothesis that the impact of *S*. *muticum v*aried among sites according to the regions because of nutrient-richer and warmer seawater in Galicia than Asturias could not be tested exhaustively, but we could expect differences between regions if thermal conditions were important.

Our findings indicated that the removal of *S*. *muticum* changed some eco-physiological traits of native species, independently of the region. The removal of *S*. *muticum* increased both gross primary production (the amount of carbon that a plant can fix in a given time) and carbon storage capacity of the primary space-holder, turf-forming *Corallina* spp. consistently across sites. It also increased the same eco-physiological traits in the canopy-forming brown seaweed *B*. *bifurcata* only at the site of Peizas. The eco-physiological traits linked to the nitrogen flow followed the same pattern, but the response was more variable and no clear patterns were identified. At Peizas site, seawater nutrient concentration was the lowest and *S*. *muticum* biomass was the greatest in spring 2012, when the labeling experiment was done. It is possible that under low seawater nutrients, the removal of a large amount of *S*. *muticum* rendered available nutrients for other competing species, like *B*. *bifurcata* [47–49]. It is therefore possible that seawater nutrients can regulate the impact of *S*. *muticum*, as expected initially, but further evidence is needed.

A laboratory experiment found that *S muticum* reduced the primary production (estimated as percentage biomass change in the experiment) of native species, including the canopy-forming brown algae *Fucus vesiculosus* Linnaeus [50]. The authors attributed this negative impact of *S*. *muticum* to shading. Canopy-forming macroalgae, like *S*. *muticum*, can successfully compete for light by forming a floating canopy that shade other underlying algae [22,47,51]. At great *S*. *muticum* biomass, as at Peizas site, the invasive species biomass could shade not only understorey, but also other canopy species, which is an alternative, non-exclusive explanation of the site-specific impact on *B*. *bifurcata*. Shading can have an intense impact on the photosynthesis of small-sized sub-canopy species of Corallinales in rock pools because individuals can have adapted metabolic processes such as ion transport or RUBISCO activity for photosynthesis to the high irradiance of low tides [52]. Some corallinales can, however, adjust their photosynthesis to low irradiance [53] and there might be other, non-exclusive mechanisms explaining the negative effect of *S*. *muticum*. For instance, daily fluctuation of pH, CO_2_ and O_2_ are particularly large under algal canopy as a result of their metabolism (e.g. photosynthesis and respiration) and mechanical alteration of water flow [49]. This may greatly impact photosynthesis and calcification of coralline-algae [54,55].

The physiological changes in *Corallina* spp. and *B*. *bifurcata* following the repeated (monthly) removal of *S*. *muticum* for over a year were not clearly reflected by changes in biomass. The apparent mismatch between physiological and growth traits may be due to the timing of the experiment. This experiment was carried out in March, at the beginning of the *S*. *muticum* growing season, which spans from January to September in this area. The large canopy is then lost through erosion and fragmentation [56]. During winter, *S*. *muticum* was therefore naturally absent from the rock pools and had only a slight effect on native species. We should also consider that, although *S*. *muticum* is regarded as an aggressive invader, its impact on native macroalgal populations and assemblages in rock pools is variable, spanning from a moderate increase or decrease to substantial changes in biomass and in species diversity [27,57,58].

Some studies have proposed that a positive effect of invasive seaweeds on ecosystem functioning (productivity, respiration or light-use efficiency) is due to the sampling effect hypothesis [14,59]. This hypothesis applied to invasion advocates that when an invasive species establishes in the assemblage, there is a high probability of including a highly productive, dominant species in the community. As an alternative, native species with a long history of co-evolution may influence ecosystem processes through resource-use efficiency [60]. Our results showed both aspects could intertwine. The negative impact of *S*. *muticum* on the gross primary production of native species was associated with their reduced carbon storage capacity and with their contribution to assemblage-level functioning, especially at the site of Peizas. The contribution of *S*. *muticum* to ecosystem functioning was particularly important at this site and counterbalanced the loss of functioning of native species, indicating *S*. *muticum* could impact resource-use efficiency. At the other sites, where there was less impact on native species, *S*. *muticum* contribution to functioning decreased according to its biomass (La Griega > Vidiago > Rocas Blancas; Fig 4), corroborating the sampling effect hypothesis of including a dominant species [14,59]. Our initial hypothesis considered the impact of the presence and not the abundance of *S*. *muticum*. The role of *S*. *muticum* biomass in determining the magnitude and direction of the impact on native species has been already recognized [27], but it should be better addressed in the context of ecosystem functioning and the management of native species control.

Invasive species are often competitively superior regarding growth or nutrient uptake [6,7]. Neither the gross primary production nor the carbon storage was greater in *S*. *muticum* than in native species. The competitive capacity of invaders may decrease once the species has become established in the remaining native assemblage. Some researchers reported that in the invasive red algae *Dasysiphonia japonic*a (Yendo) H.-S. Kim, the efficiency of taking up nutrients decreased progressively during the process of invading a new community. When established, the invasive species reached an efficiency level close to that of the remaining native species [10]. In some areas of Portugal, where *S muticum* has established since the 1980s, nitrogen uptake efficiency was found to be higher in the canopy-forming native species *Cystoseira humilis* Schousboe ex Kuntzing than in *S*. *muticum* [61].

## Conclusions

Biological invasions can modify native communities and ecosystems through complex patterns, acting first on the physiology and survival of individual organisms and, in turn, propagate their effects to community structure and ecosystem functioning through interactions with the environmental setting at different biological scales [3,16,51]. We showed that the impact of *S*. *muticum* on the eco-physiology of native seaweeds propagated to assemblage-level functioning and that at the time of the year when the experiment was done the thermal conditions were unlikely to play a role in shaping native assemblage response. Invasive species biomass and nutrient availability might be important, but we need further evidence. Interestingly, we found that when the invasive species was removed, the remaining native species could recover their functional role, which might have implication for managing the control of established invasive species. Although forecasting invasion impact on ecosystem functioning is a major focus of ecological research and ecosystem management, we still lack knowledge about how physiological processes and species interactions may cause changes in ecosystem functioning. Our study findings highlighted the importance of including eco-physiological response to invasion for understanding, anticipating and perhaps mitigating the impacts of invasion on ecosystem functioning.

## Supporting information

S1 Fig Temperature and nutrient concentration. Average monthly values of seawater temperature at high tide (n = 60) between November 2010 and September 2011 and seawater inorganic nutrients (n = 4) from autumn 2010 to spring 2012.
(TIF)

S2 Fig Fluctuation of excess stable isotopes (E, as described in Eq 2) in macroalgal thalli during the 4 days after the end of the incubation period. Mean (SE) % atoms of ^13^C (a) and of ^15^N (b) in native macroalgae and in the invader *S*. *muticum*.
(TIFF)

S3 Fig Two-dimension non-metric multi dimensional scaling (nMDS). The ordination is based on the triangular matrix derived from the Bray-Curtis index measuring differences in native species composition between macroalgal assemblages from control (+S) and *S*. *muticum—*removed rock pools (-S) at each of the four sites indicated in the graph. P = Peizas, RB = Rocas Blancas, LG = La Griega and V = Vidiago.
(TIF)
