# Supplementary figures and images for: Removal of an established invader can change gross primary production of native macroalgae and alter carbon flow in intertidal rock pools
Source: PLoS One. 2019 Dec 3;14(12):e0217121. doi: 10.1371/journal.pone.0217121 (PMC6890258; doi:10.1371/journal.pone.0217121)

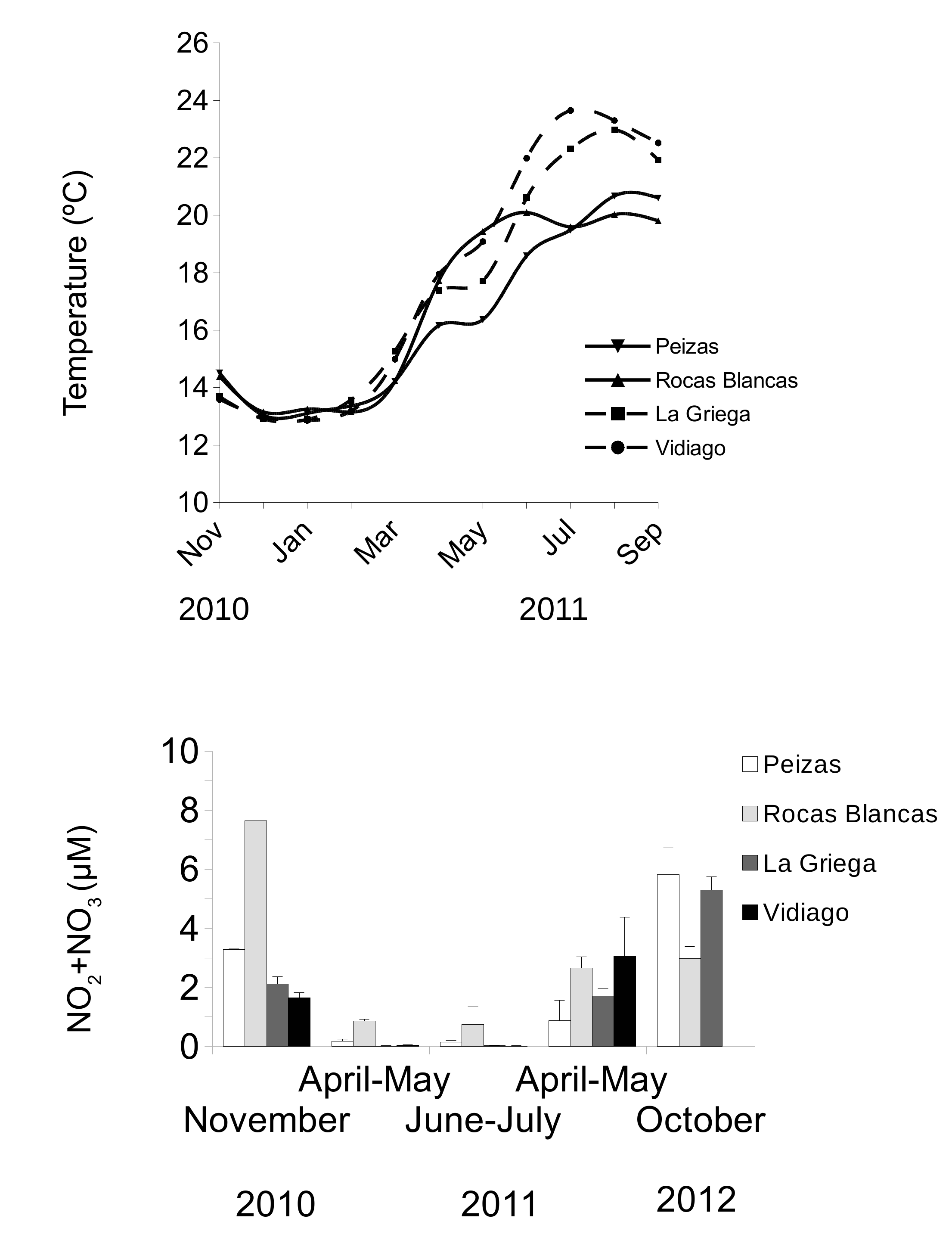

Supplement: S1 Fig — Average monthly values of seawater temperature at high tide (n = 60) between November 2010 and September 2011 and seawater inorganic nutrients (n = 4) from autumn 2010 to spring 2012. (TIF) [file pone.0217121.s001.tif]

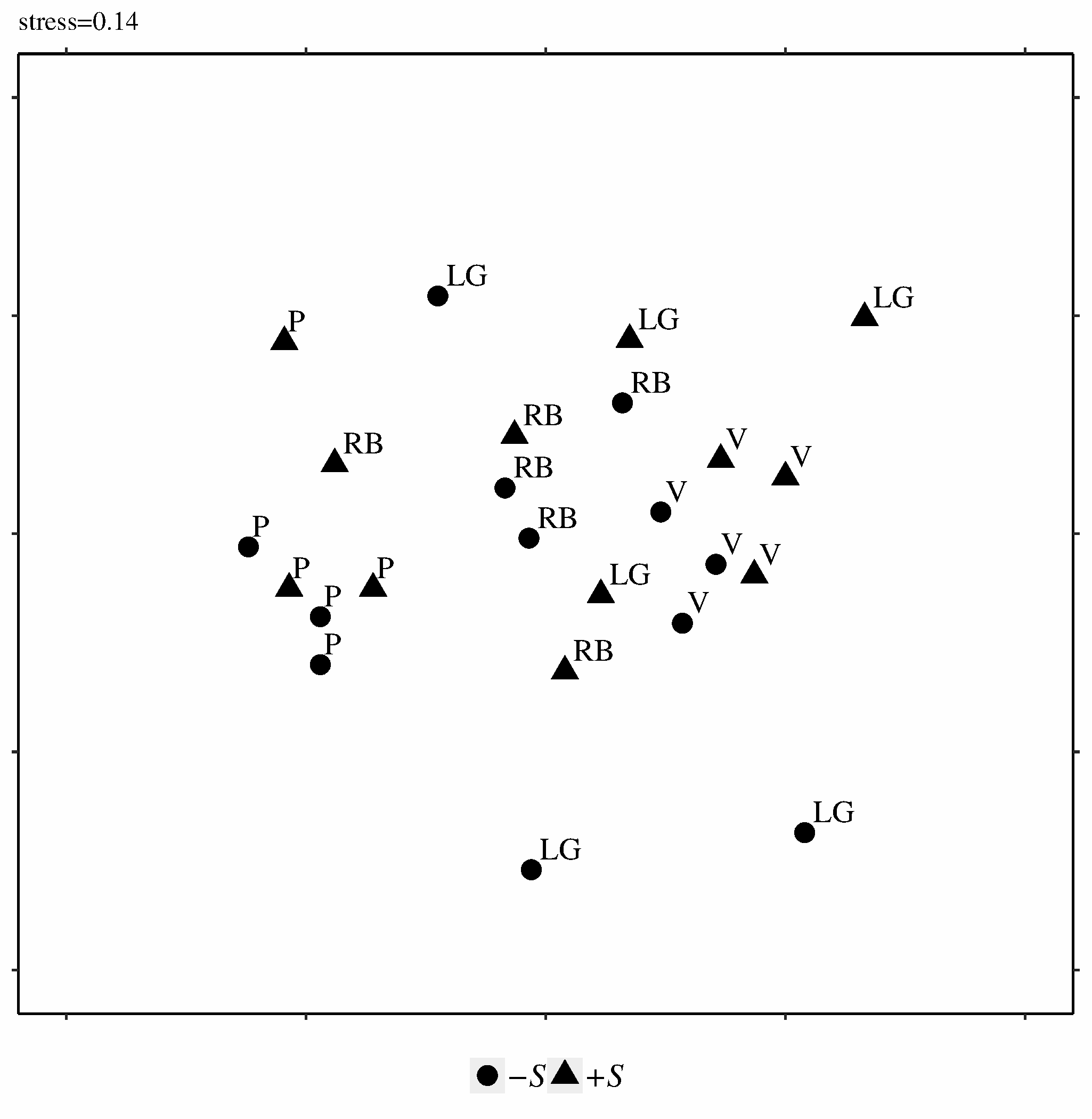

Supplement: S3 Fig — The ordination is based on the triangular matrix derived from the Bray-Curtis index measuring differences in native species composition between macroalgal assemblages from control (+S) and S. muticum—removed rock pools (-S) at each of the four sites indicated in the graph. P = Peizas, RB = Rocas Blancas, LG = La Griega and V = Vidiago. (TIF) [file pone.0217121.s003.tif]
